# The DISCOURSE in psychosis (London Ontario): A speech dataset to examine communication disturbances in early-stage psychosis

**DOI:** 10.1016/j.dib.2026.112517

**Published:** 2026-01-27

**Authors:** Brian Cho, Estée Balles, Michael Mackinley, Paulina Dzialoszynski, Sabrina Ford, Rohit Lodhi, Lena Palaniyappan

**Affiliations:** a Schulich School of Medicine and Dentistry, Western University, London, Ontario, Canada; b Robarts Research Institute, Schulich School of Medicine and Dentistry, Western University, London, Ontario, Canada; c Douglas Mental Health University Institute, McGill University, Montreal, Quebec, Canada; d Department of Psychiatry, Western University, London, Ontario, Canada

**Keywords:** Schizophrenia, Natural language processing, Language models, Voice, Digital health

## Abstract

Advances in speech technology and Natural Language Processing (NLP) have demonstrated promise in using speech as a valid source of data to detect features of psychosis. These technologies can potentially detect subtle speech aberrations that often go unnoticed by clinicians and family members. However, research in this area is hindered by a significant limitation: a lack of sufficient and appropriate speech corpora from psychosis patients, especially datasets containing naturalistic speech that reflects typical clinical interactions. This scarcity limits the development, testing, and generalization of new computational methods for psychosis prediction. To address this gap, our new dataset offers naturalistic speech samples collected using the semi-structured DISCOURSE protocol. This resource includes both raw audio recordings and transcribed speech from individuals participating in an early-stage psychosis treatment program (<5 years of illness), alongside demographically matched healthy controls, in English. In addition to speech data, the dataset provides comprehensive clinical, cognitive, and demographic information for each participant. Importantly, the DISCOURSE protocol and clinical assessments were repeated after a 12-month follow-up to assess stability and change in speech, symptom burden and functional status. As the inaugural dataset released by the DISCOURSE consortium, this resource marks the beginning of a series of harmonized data collection efforts across multiple countries and languages. This multi-site, multi-language approach enables validation of findings in diverse psychosis populations, allowing researchers to address questions that cannot be resolved at individual research sites. Transcripts were extracted from conversations lasting between 15 and 35 minutes in total. This data herein can be used to perform analyses on acoustic, semantic, syntactic and pragmatic measures related to psychosis, as well as in understanding the nature of communication difficulties faced by patients. We expect this dataset to be useful for future investigations into speech data’s clinical utility in assessing thought disorder and psychosis-related symptoms.

Specifications Table

**Table:** 

Subject	Health Sciences, Medical Sciences & Pharmacology
Specific subject area	Speech, thought, language and communication in patients with psychosis.
Type of data	Audio files (.mp3 format) Transcription (.CLAN format) Table (.csv format)
Data collection	The data was collected by: (1) Standardised questionnaires to gather clinical (10 items of PANSS) and cognitive data; (2) A semi-structured DISCOURSE protocol to elicit speech across 7 contexts, with the full interview recorded using an Olympus/SONY voice recorder; (3) Transcriptions generated by using the Batchalign Python program in the CHAT/CLAN system through PsychosisBank. The data was collected between November 2021 and April 2025. Participant were interviewed over 20-minutes continuously in a quiet room with minimal distractions, with a repeat assessment completed one year later.
Data source location	The data were collected in London Ontario, and the curated data is stored in the PsychosisBank.
Data accessibility	Repository name: PsychosisBank Direct URL to data: https://talkbank.org/psychosis/access/English/Palaniyappan/Discourse-UWO.html Instructions to Access Data: Access to the Data is granted by the DISCOURSE consortium Data Access Committee to approved researchers (No access will be granted for commercial purposes). For complete instructions on becoming a Psychosisbank member please see: https://talkbank.org/psychosis/membership.html
Related research article	Melshin G, DiMaggio A, Zeramdini N, MacKinley M, Palaniyappan L, Voppel A. Taking a look at your speech: identifying diagnostic status and negative symptoms of psychosis using convolutional neural networks. NPP—Digital Psychiatry and Neuroscience. 2025 Jul 8;3(1):19.

## 1 Value of the Data

• The data provides speech obtained in a naturalistic face-to-face interaction with the interviewer with various stimuli and prompts. This enables the study of the effect of psychosis on communication with varying degree of cognitive demands. Researchers can use these data to develop, train, and robustly validate computational models that detect subtle linguistic and acoustic features associated with psychosis, thus advancing the potential for speech to serve as a biosocial marker for identifying diagnostic status.
• This dataset also provides clinical and cognitive measures, including rating-scale scores of thought disorder, collected at the same time as speech recordings. This allows construct validation of linguistic variables in psychosis.
• With repeat assessment after a 12-month follow-up using the same data collection protocol, this dataset enables assessment of changes in speech and language in relation to symptom progression.
• The dataset includes audio files and transcripts that allow for multi-level analyses (acoustic, semantic, syntactic, and pragmatic), for a deep-phenotypic characterisation of speech patterns in psychosis.

## 2 Background

A large body of literature has supported the core role of language disturbance in psychosis as a predictor of illness onset [[1], [2], [3]], persistent disability [4,5], poor response to treatment [6,7] and reduced quality of life [8]. In recent times, a surge in quantitative approaches[9] to process linguistic readouts has expanded the methods of inquiry in the field [[10], [11], [12]]. Along with a move towards Big Data approach for deep phenotyping, these advances blend well with the increasing interest in offering measurement-based care in an era of digital health.

There is a growing need to harmonize data collection for future studies to enable data pooling, comparative linguistics and improve the efficiency of focussed enquiries [13]. Standardizing the acquisition, transcription, and processing of automated speech analysis is therefore a key priority.

Corpus-based computational linguistics can be leveraged to address 4 key domains of research: 1) refining the phenomenology of thought disorder, 2) developing predictive and monitoring applications leveraging speech, 3) in deep phenotyping language in psychosis, 4) in ascertaining key markers of communicative behaviour that relate to core psychopathology.

## 3 Data Description

The files included in this dataset are:

1. Audio files in .mp3 format of people with established psychotic disorders and health controls of the full DISCOURSE protocol (unstructured conversation, personal narrative, picture description [3 TAT pictures], health narrative, dream reports, storyboard [lighthouse], reading and recall [pitcher and crow story]), from both baseline and 1-year follow-up, recorded in English.
2. Transcription of the audio files in .clan format of the full DISCOURSE protocol (unstructured conversation, personal narrative, picture description [3 TAT pictures], health narrative, dream reports, storyboard [lighthouse], reading and recall [pitcher and crow story]), from both baseline and 1-year follow-up, written in English.
3. DISCOURSE-based demographic data of patients and health controls on consensus diagnosis, gender, hospital status, education, employment, education/training, job, language, ethnicity, and immigration status. The data also includes clinical data of self-endorsed substance use, psychotropic drugs, symptom severity in schizophrenia, occupational and educational functioning data, and occurrence of relapses over 1 year. Additionally, some of the samples have clinical scores for verbal fluency and modified digit symbol substitution; these data were collected starting partway through the project, so they are not available for every participant. This cognitive data is available for N=79 of our patient population, and N=30 of our healthy controls.

Sociodemographic Descriptions

**Table:** 

Sociodemographic Factor	Classifications
Patient Category	Healthy Control, Patient
Consensus Diagnosis	Schizophrenia, Schizoaffective, Bipolar Disorder with psychotic features, Psychosis NOS
Gender	Female, Male
Hospital Status	Outpatient, Inpatient, HC
Education	≤Gr9, ≤Gr 12, HS, College/Vocational, BA/BSC, MA/MSC/PhD
Employment, Education or Training	Yes, No
Job	Higher Occupation/Professional, Intermediate/Management, Self-employed/Own account, Lower supervisory/Technical, Semi routine/routine
Language at Home	English, French, Spanish, etc
Language Neighbourhood	English, French, Spanish, etc
Language at School	English, French, Spanish, etc
Ethnicity (Self-Identified)	Caucasian, Indigenous/First Nation, Hispanic, Black, Middle Eastern, Mixed Race
Immigration Status	Canadian born, Outside Canada

Cognitive and Clinical Scores

**Table:** 

Metric	Definition
Alcohol Use	Measures of AUDIT-C
Smoking	Smoking Index (cigarette/day x years of tobacco use)/20
Cannabis Use	Cannabis Abuse Screening Test (CAST)
Substance Use Questionnaire (SUQ)	Self-reported use of alcohol and other recreational substances
Antipsychotic Medication	Dose, medication, length or treatment, along with other medication use
Clinical symptom severity in schizophrenia	Positive and Negative Syndrome Scale (PANSS)
Assessment of social and occupational functioning	Social and Occupational Functioning Assessment Scale (SOFAS)
Category Fluency Score	Test of semantic verbal fluency
Modified Digit Symbol Substitution Test	Assessment of cognition for processing speed
Thought Language Index	Instrument for assessing formal thought disorder (metrics of both impoverishment and disorganization) based on a picture description task.

1 Year Follow-Up Data

**Table:** 

Hospitalizations	Patient chart review to assess for inpatient stays and length of stay over the past year
Medication Changes	Psychotropic medication changes in dose and type
Relapse Judgement	Questionnaire to assess potential relapses over the past year

## 4 Experimental Design, Materials and Methods

### 4.1 Participants

109 patients were recruited from the Prevention and Early Intervention Program for Psychosis in London Ontario, along with 60 health controls. The participants were 18-50 years old, met the operational criteria for a psychosis disorder as per the Diagnostic and Statistical Manual of Mental Disorders (DSM) 5^th^ edition criterion, and were diagnosed with a psychotic disorder by a psychiatrist. Healthy controls were recruited through word of mouth, social media, and community posters from the same geographic location where patients were recruited from. Compensation was not advertised as a recruitment incentive as per the ethical approval, however, participants were provided up to $50.00 CDN to cover their inconvenience. Following the consensus diagnosis procedure, of our patient cohort (N=109) n=58 had a diagnosis of Schizophrenia, n=24 had a diagnosis of Schizoaffective, n=22 had a diagnosis of Psychosis NOS, n=5 had a diagnosis of Bipolar Disorder with Psychotic Features. These diagnoses were confirmed by the research team at both points of contact.

Those with psychosis secondary to a Substance Use Disorder or with an active dependence pattern in the past year, or a known neurological disorder impacting speech output (e.g., apraxia) were excluded.

**Table:** 

Variable	First Episode Patients: N=109	Healthy Controls: N=60
Sex (M/F)	83 / 26	38 / 22
Age at baseline [m (SD)]	28.59 (7.59)	28.23 (7.81)
NS-SEC [m (SD)]	2.03 (1.10)	3.52 (1.51)
Education [m (SD)]	4.68 (1.12)	3.51 (0.93)
PANSS-10 item total [m (SD)]	20.11 (7.55)	10.00 (0.00)
Returned for 12 month follow up # (%)	92 (84.40%)	52 (86.66%)

Note: NSSEC: Parental socioeconomic data based on National Statistics - Socioeconomic Classification of Rose and Pevalin; PANSS-10: 10 items of Positive and Negative Symptoms Scale.

### 4.2 Experimental procedure

Participants had 2 visits: baseline visit, and at 1-year follow-up.

Baseline visit

*Patient Interview*

Following the completion of all consent procedures, The Social and Occupational Functioning Assessment Scale (SOFAS), Substance Use Questionnaire (SUQ), Positive and Negative Syndrome Scale (PANSS), Category Fluency Test, and modified Digital Symbol Substitution Test were administered.

*DISCOURSE Protocol*

Speech tasks following a detailed script were administered in English. The script and instructions for administration are available from the website discourseinpsychosis.org. There were 7 sections, with 2 interviewer prompts/tasks for each section. If participants had a pause greater than 10 seconds, or failed to initiate speech output for 30 seconds, prompts such as “can you tell me more”; “anything else?” were used to encourage their speech.

Section 1 involves participants describing themselves, e.g., what they did for work or school. The rationale was to begin the session with topics that were personally familiar and appropriate for the individuals age and culture, without challenging their learned knowledge.

Section 2 involves asking the participants about an important event that happened in their life. If they were unable to respond, they were asked about how their last week has been. The rationale was to create a linguistic window to autobiographical narratives with a personal perspective.

Section 3 involves asking the participants about their mental health; e.g., do they have a mental health issue, what they think it is, how it started, how this has affected their life and how they feel about living with it. The rationale for this section was to gather symptom-related content, as well as an insight-oriented reflective narrative [14]. This section is usually short for healthy subjects that endorse no mental illnesses or treatments.

Section 4 involves the participants describing 3 standardized pictures derived from the Thematic Apperception Test for 3 minutes (1 minute per picture). The interviewers were instructed to elicit no more than a maximum of 2 minutes per picture. The rationale behind this section was to gather a narrative around a visually-grounded external referent, with multiple descriptive components [15].

Section 5 involves asking the participant to describe the central events of a 6-panel story board that is without text. This also provided data on a narrative with external focus, but invoked descriptions of another person’s mental state, that is independent of one's personal experience [16].

Section 6 involves asking of their recent dream or if they had any dreams that repeat and to describe one of them. The rationale behind this was to gather rich descriptions of an event that the participant knows as unreal [17].

Section 7 involves the participant reading aloud a one-page short story. Then, they are asked to recall the story in their own words. This provides articulatory information for a specific text, as well as a comparison with ground truth text when performing the immediate recall. Such narrative recall tasks have been successfully employed to study connected speech in neurocognitive disorders such as dementia [18,19]. More detailed information on administering this protocol is available on the DISCOURSE website with demonstrative videos.

*Follow-up*

At the 1-year follow-up, the same procedures from the initial visit was repeated. Additionally, a relapse checklist based on clinical information was completed retrospectively for patients based on information from medical records (hospitalizations / emergency visits and change of care) over the last year.

### 4.3 Data collection

The speech samples were recorded with an Olympus VN-541PC 4GB Digital Voice Recorder (V405281BU000) and a SONY Stereo Digital Voice Recorder with Built-in USB, 4 GB (ICDPX470). The recorder was positioned centrally on an assessment table less than 4 feet from both research staff and the participant to ensure that the discourse could be captured from both speakers.

### 4.4 Data processing

Transcriptions were automatically generated with Batchalign, a Python program that can create CHAT transcripts from recorded audio files [20]. The generated transcripts were then manually verified by researchers with the original audio recordings using the CLAN program [20]. Original audio files and the verified transcripts were then uploaded to PsychosisBank servers. Of note, as audio files are available, the transcripts can be regenerated by researchers interested in alternate ways of processing the data.

## Limitations

While the data provides many avenues for investigation, we report several limitations. First, despite extensive training prior to processing the data, slight variation in prompting behaviour between research staff may be present given the naturalistic paradigm that was employed. The dataset also lacks cognitive scores from the first 60 baseline assessments as this was added after a protocol amendment approved by the REB. Finally, as recruitment of patients was done largely within a first-episode psychosis clinic, our sample is likely biased to patients who are actively engaged in treatment and may not reflect the full spectrum speech in psychosis.

## Ethics statement

Written informed consent was obtained from all patients/participants prior to participation to the study. Research Ethics Board at Western University approved all study procedures.

## CRediT Author Statement

**Brian Cho**: Data curation, writing - original draft preparation. **Estée Balles**: Data curation. **Michael Mackinley**: Investigation, data curation, writing - review, & editing, project administration. **Pualina Dzialoszynski**: Investigation, Data Curation. **Sabrina Ford**: Investigation, Data Curation. **Rohit Lodhi**: Project Administration. **Lena Palaniyappan**: Conceptualization, methodology, software, writing - review & editing, supervision, project administration.

## Data Availability

PsychosisBankDISCOURSE in Psychosis - UWO Data (Original data).
